# Supplementary material for: BocODD1 and BocODD2 Regulate the Biosynthesis of Progoitrin Glucosinolate in Chinese Kale
Source: Int J Mol Sci. 2022 Nov 26;23(23):14781. doi: 10.3390/ijms232314781 (PMC9739482; doi:10.3390/ijms232314781)
Supplement: Supplementary file 1 [file ijms-23-14781-s001.zip › ijms-2018874-supplementary.pdf]

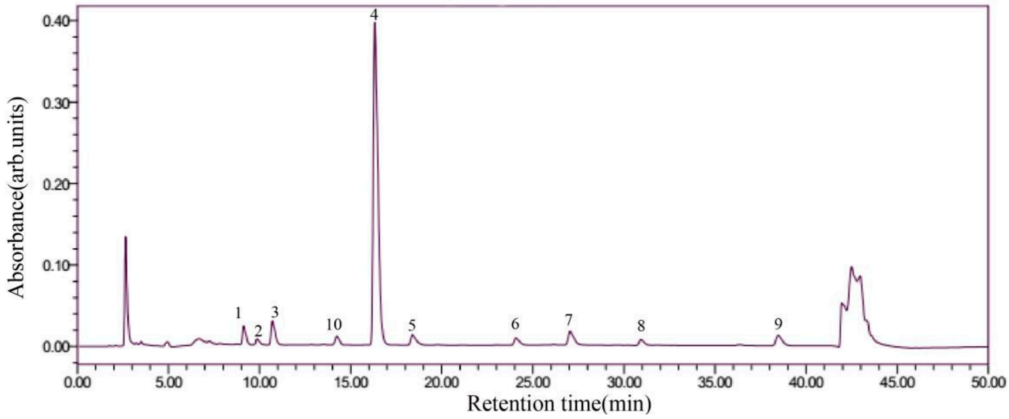

Figure S1: HPLC chromatogram of desulfoglucosinolates. 1. 2-hydroxybut-3-enyl desulfoglucosinolate; 2. 4-(methylsulfinyl)butyl desulfoglucosinolate; 3. 2-propenyl desulfoglucosinolate; 4. but-3-enyl desulfoglucosinolate; 5. 4-hydroxyindol-3-ylmethyl desulfoglucosinolate; 6. 4-pentenyl desulfoglucosinolate; 7. indol-3-ylmethyl desulfoglucosinolate; 8. 4-methoxyindol-3-ylmethyl desulfoglucosinolate; 9. 1-methoxyindol-3-ylmethyl desulfoglucosinolate.

Table S1: The primer list

Primer name	Primer sequence
Actin 2	Forward :5′-GAGGCTCCTCTTAACCCAAA-3′
(qRT-PCR)	Reversed:5′-CAGAATCAAGCACAATACCG-3′
BocODD1	Forward:5′-GGAGTCAAACTGCGTAATGG-3′
(qRT-PCR)	Reversed:5′-TATGCGTGGGATTTTTGTGA-3′
BocODD2	Forward:5′-ACTCAAGAAAGTAAAGCAGG-3′
(qRT-PCR)	Reversed:5-GAGCGGGATTTTTTTGATTC-3′
BocODD1	Forward:5′-GGACTCTAGAGGATCCATGGCGGGAAGATTCGACCG-3′
(over-expression)	Reversed:5′-GGGGAAATTCGAGCTCTCAGATCCTTAAATGGCTCA-3′
BocODD2	Forward:5′-GGACTCTAGAGGATCCATGTCGGGAACATTTGACCG-3′
(over-expression)	Reversed:5′-GGGGAAATTCGAGCTCTCAGATCCTTAAATGGCTCA-3′
BocODD1/2	Forward:5′-CGGATTTAAATGCAAGATCCAGAAGCGAGGA-3′
(RNAi-positive)	Reversed:5′-CCGCCATGGGCTCAGGACACCGCGGGTAA-3′
BocODD1/2	Forward:5′-CATGGATCCGCAAGATCCAGAAGCGAGGA-3′
(RNAi-negative)	Reversed:5′-CTACCCGGGGCTCAGGACACCGCGGGTAA-3′

Table S2. Analysis of physicochemical properties of genes

No	Gene	Molecular weight (KDa)	Therotical pI	Instability index	Aliphatic index	GRAVY
1	*AtODD (At2g25450)*	40351.27	5.86	40.10(>40) unstable	81.42	-0.281
2	*BocODD1*	40230.23	6.87	32.94(<40) stable	83.6	-0.367
3	*BocODD2*	40182.08	6.19	32.45(<40) stable	80.06	-0.395
4	*BocODD3*	30122.56	5.27	36.82(<40) stable	77.37	-0.395
5	*BocODD4*	40936.86	5.61	37.83(<40) stable	83.91	-0.299

Suplementary Date1: Sequences information of four BocODD genes

>BocODD1 promoter sequence:

agatattgtaacatttcacaaattggaaagtttttaaaaaattaaacttttcgcttcata
aatttatactatcgagtaaataattaaatatttagtttttgtttaatttttaaaataaac
tatatagtttaaaatttgttttcattggtttaaggtagtaaagattaatcattgttagat
aatatgatttttgttatttaaaaaaatctttataattttaaaagttaacatcgataaata
tttaaatatttaacatatagaagtatagtattacaacattaaattatatatatttaattt
atattatctataaatccaatggattatctattgtttaaatccaattattgatagcccaat
aaaaatttctggtagacccaaaatttaaatgataagattagagattaaatgtaacatgac
tttatatgaataggtccattgagtctatttttttaaaaaatcacacatgaatcaaagttat
gacttctattttaatatataagatatattttttttgtccaaagcccttttatagtaacga
gtcagccctaatagaaagtcacatgcttttctatctcggaccagatctcattttgttagc
attacctatttaaacaatttatgcatcatggttaacaaaatatagatcagtgaaagttta
caattgaaaatctattatataagtcagtttttctatgttgttcttatattggttttacaa
ttaatgattatgatatataacgaaaatgttatatgttgacaaaaaactaaaataatatat
ataatgttattataaaaaatattttcaatttacataatagtttttcaattaaaattttag
gtttagtttcatttttttagttttcccaaaccatatgtaataaaaatggaaaatatttat
agaaaattcttgaaattgcatttttacttattattgctgacatttataacttttaaatca
catcaattccaaagatgcaccgcgctttggtttccattcataatataaaaatgatttatt
aatatgataaattattgataatatgatatattattgataatatgataaaataattataac
taggcctaaaagatacacatctcgatgataagataattatttttaataataggagtatga
tataatattgatgatctctttttgacattaataatactgacgatatcttaatatatctcc

ttggttctctgtcatccacgttcaacccttattaattaatgttaaccttttttaatccaa
ttagtggcttcaggttaactactacgaaatgatatcagcaaattactatacttggaatat
cagaacatctttcaagtttccagtataataagttaagtaaggttaatacactaataaatg
gaagaaaatataaacacgatttgataaacaaagatacaaatttcatccagaagaatttac
ctagctctccaaaccaaaaaacaaaaaaaaaattacctagctctccaaagaagaataata
aaccttagtcatatgcttgtgccacccaaatataataaaataaagataaattgctgaatt
tgatgcacaacaacgacaatacttcggaaaatcatgttcgagtaattagatagtcgattg
tgcattgttattaatttgacttttgtaaagaaaaaaaattatcctattacagctttgagtg
atatccattcttttagcataatatttttcttgggtaagtatgatctatttataagatata
caaatcgccctagcgtttactctaaatctcactttctgaagcttaaatgactcttttcta
aaccaaattttagagtattattttttatgtattggcacgcgcgccgctgaatggattgat
tacacgacccgtccttctctttggcctctatctcatgccttcaccagttttccttcctta
taggtgactcaaaacacaacacaagtttctctctttcctttcataaaacactcaaaaaaa
agttggagtcaaactgcgtaaATGGCGGGAAGATTCGACCGTGCGGGTGAGGTAAAAGCA
TTCGACGAGATGAAAATCGGCGTGAAGGGTCTAGTCGACAACGGAATCACAAAAATCCCA
CGCATATTCCATAACCCGCAAGCCACAATAACTAACCCTAAACCTCCTTCTACCTTGACT
ATCCCAACGATAGATCTCGGAGGTGGCGTGTTCGAGTCCACGGTCACAAGGAAGGAAGTG
ACTGAGAAGGTGAAAGGCGCCATGGAGAAGTTTGGTTTCTTCCAGGCGATAAATCATGGG
ATTCCACTCGAGGTCTTGGAGAAGATGAAAGATGGGATCCGTGCGTTTCACGCGCAAGAT
CCAGAAGCGAGGAAAAGGTTCTATAGCCGTGAAAAAACCAAAGCGATTAAGTATAACTCT
AACTCTGATCTCTATGACTCTCCTGCTGCGAGCTGGAGAGATACCTTAAGTTGTTTTATG
TTCCCTGATGTTCCCAAAACCGATGACTTACCAGACATTTGTAGGTACGAACACGGCTTC
TCTTGTTTTAAATTAATCTTTGGACTTACTTAATATTTTTGTTTGATGGGTTATGAGAGT
TTATTTATGTTGAATGTTTTAGGGAGATCATGTTGGACTACTCAAAGAGAGTGATGATGT
TTGGGGAGTTAATATTTGAGCTTATATCAGAATCCTTAGGGCTGAAGCCTAACCACCTCA
AAGAAATGGATTGTGCAAAAGGCTTGTTGATGCTCTGTCATTGTTACCCGCGGTGTCCTG
AGCCAGACCTAACGCTCGGCGCCACTCAGCATACAGACAGATCTTTCATCACTATACTTC
TTCAAGACCATATTGGAGGACTTCAAGTTCTCCATGATGGATACTGGATCGATGTTCCTC
CTAATCCTAATGCTCTTATCCTTAATGTTGGAGATCTCCTACAGGCAAGTTGTTGTTTAC
ACTTATAATCAAACGGTCCATAATTAGTAAGAAAACAAGGTCTTCATAGTGTTTTTTTTT
TTTTGCAGCTTATAACGAATGACAAGTTTGTGAGTGTGGAGCATAGAGTTTTGGCAAATG
GAGGTAAAGAGCCACGCACTTCGGTTGCATCTTTCTTTGTGCATCCTCCTTCAATAAGTC
CGAGAGTATATGGACCCATTAAAGAGCTTTTGTCTGAAGAAAACCCTCCCAAGTACAGGG
AAACCACTCCGGAAGCCTCCAACCACTATGTGGCTAGAAAACGTGATGGGAACAATTCGT
TGAGCCATTTAAGGATCTGA
>BocODD1 DNA sequence:
ATGGCGGGAAGATTCGACCGTGCGGGTGAGGTAAAAGCATTCGACGAGATGAAAATCGGCGTGAAGGGTC
TAGTCGACAACGGAATCACAAAAATCCCACGCATATTCCATAACCCGCAAGCCACAATAACTAACCCTAAACC
TCCTTCTACCTTGACTATCCCAACGATAGATCTCGGAGGTGGCGTGTTCGAGTCCACGGTCACAAGGAAGGA
AGTGACTGAGAAGGTGAAAGGCGCCATGGAGAAGTTTGGTTTCTTCCAGGCGATAAATCATGGGATTCCAC
TCGAGGTCTTGGAGAAGATGAAAGATGGGATCCGTGCGTTTCACGCGCAAGATCCAGAAGCGAGGAAAAG
GTTCTATAGCCGTGAAAAAACCAAAGCGATTAAGTATAACTCTAACTCTGATCTCTATGACTCTCCTGCTGCGA
GCTGGAGAGATACCTTAAGTTGTTTTATGTTCCCTGATGTTCCCAAAACCGATGACTTACCAGACATTTGTAG
GTACGAACACGGCTTCTCTTGTTTTAAATTAATCTTTGGACTTACTTAATATTTTTGTTTGATGGGTTATGAGA

GTTTATTTATGTTGAATGTTTTAGGGAGATCATGTTGGACTACTCAAAGAGAGTGATGATGTTTGGGGAGTTA
ATATTTGAGCTTATATCAGAATCCTTAGGGCTGAAGCCTAACCACCTCAAAGAAATGGATTGTGCAAAAGGCT
TGTTGATGCTCTGTCATTGTTACCCGCGGTGTCCTGAGCCAGACCTAACGCTCGGCGCCACTCAGCATACAG
ACAGATCTTTCATCACTATACTTCTTCAAGACCATATTGGAGGACTTCAAGTTCTCCATGATGGATACTGGATC
GATGTTCCTCCTAATCCTAATGCTCTTATCCTTAATGTTGGAGATCTCCTACAGGCAAGTTGTTGTTTACACTTA
TAATCAAACGGTCCATAATTAGTAAGAAAACAAGGTCTTCATAGTGTTTTTTTTTTTTTGCAGCTTATAACGAA
TGACAAGTTTGTGAGTGTGGAGCATAGAGTTTTGGCAAATGGAGGTAAAGAGCCACGCACTTCGGTTGCAT
CTTTCTTTGTGCATCCTCCTTCAATAAGTCCGAGAGTATATGGACCCATTAAAGAGCTTTTGTCTGAAGAAAA
CCCTCCCAAGTACAGGGAAACCACTCCGGAAGCCTCCAACCACTATGTGGCTAGAAAACGTGATGGGAACA
ATTCGTTGAGCCATTTAAGGATCTGA

>BocODD1 cDNA sequence:

ATGGCGGGAAGATTCGACCGTGCGGGTGAGGTAAAAGCATTCGACGAGATGAAAATCGGCGTGAAGGGTC
TAGTCGACAACGGAATCACAAAAATCCCACGCATATTCCATAACCCGCAAGCCACAATAACTAACCCTAAACC
TCCTTCTACCTTGACTATCCCAACGATAGATCTCGGAGGTGGCGTGTTCGAGTCCACGGTCACAAGGAAGGA
AGTGACTGAGAAGGTGAAAGGCGCCATGGAGAAGTTTGGTTTCTTCCAGGCGATAAATCATGGGATTCCAC
TCGAGGTCTTGGAGAAGATGAAAGATGGGATCCGTGCGTTTCACGCGCAAGATCCAGAAGCGAGGAAAAG
GTTCTATAGCCGTGAAAAAACCAAAGCGATTAAGTATAACTCTAACTCTGATCTCTATGACTCTCCTGCTGCGA
GCTGGAGAGATACCTTAAGTTGTTTTATGTTCCCTGATGTTCCCAAAACCGATGACTTACCAGACATTTGTAG
GGAGATCATGTTGGACTACTCAAAGAGAGTGATGATGTTTGGGGAGTTAATATTTGAGCTTATATCAGAATCC
TTAGGGCTGAAGCCTAACCACCTCAAAGAAATGGATTGTGCAAAAGGCTTGTTGATGCTCTGTCATTGTTAC
CCGCGGTGTCCTGAGCCAGACCTAACGCTCGGCGCCACTCAGCATACAGACAGATCTTTCATCACTATACTTC
TTCAAGACCATATTGGAGGACTTCAAGTTCTCCATGATGGATACTGGATCGATGTTCCTCCTAATCCTAATGCT
CTTATCCTTAATGTTGGAGATCTCCTACAGCTTATAACGAATGACAAGTTTGTGAGTGTGGAGCATAGAGTTT
TGGCAAATGGAGGTAAAGAGCCACGCACTTCGGTTGCATCTTTCTTTGTGCATCCTCCTTCAATAAGTCCGA
GAGTATATGGACCCATTAAAGAGCTTTTGTCTGAAGAAAACCCTCCCAAGTACAGGGAAACCACTCCGGAA
GCCTCCAACCACTATGTGGCTAGAAAACGTGATGGGAACAATTCGTTGAGCCATTTAAGGATCTGA

>BocODD2 promoter sequence:

gaggccacaaatccggaatgggagtccttgttgaggaacatgcgacgacaaaatcccatt
ccaggtgagtcatcatccgacacacatcccgaggcggatgtagagaggaggagtaatgaa
atctaccgggcgatgaacgactcttagtttatttttcggttgttgaattataaattcaa
aacttatttatatataaaatattttggtattgatttttttaaaaaataattttattaat
aaattaaataattttaattatttttattaattatatttttaaattctgtaaaataaaaaa
atgaagtaaattcgtagctaatttactacctatttacgtggaaactttacgaggaaatga
cgagaaaaaataaacgagtattttacgaggaaacgtaaacgaggaaatgaagaagaaaga
taaacgagtattttacgagcaaatactttcgtggtagttgcgtgtactttacgaggaaac
actttcgagatatttacgtgtagtttacgaggaacacgtttcgaggtatttacgaggaaa
tatagcgagctccttacgtggaatgtttgcgtggtctttacgacgaaatgatgtacttcg
tctttacgactaaatatttttctcgctaagttacgacgaattggcgagaaaatatgtgtt
acgacgaacgagtaacgacgaaactcgtttcctcgctaattcctcgtaaaacctttctta
cgacgaactcacgaggaaaaccgccctcgttaaacttatgttttcttgtattgttaaatg
tagctttatttatttaactatctaccaaaggtataaatatttacataattttcaatttta
aaataataatgttttactattttatttcttcttaaaattaaatttaacaaattttacttt
tgttggttttaatttaatatatttaacttataaattttttatttaacgtaaagagtcta

atgctaattactcatttgaattattttactgtaactaaattttaatacattcaacaattt
agaaatttgattaggtttatcacttaaatgtagctttatttatttaactatctacaaaag
gtataaatatttacataattttatctttgatatgtcataacaattattaaaatgtctatt
tttatgttaaaatttaaatatcaataataaatcgatcatgttacaataattttaacatca
tgtaatgacctttgaaaataacttgatattatttataaaaaaatatgataaggacacaat
ttttttaaaaaatatgatattgtttgaaatatgtttttgtaaaataaataaaatttgca
aataagaagcacatatttttttagcaaaaaaattatttgcagattttaaatacaataaatg
ttataaaaagcactttttatttagatatgataattcatattctggtattttatgtcttaa
ttatagtatcatgtatatacttttgtaaaacaaattcaaaaatataaaaagtatataaaa
caatttaattaagaaaatataagtattagtaatgaaatatattcttacaattttaccata
tatatatatggatgacttctcattagaaacatagaaaatatataaaatacaaataaaata
aatgaatattttaattaattaatttaatttgtttagtgtgcaagactgcaagtttataat
attattaataataattacgatactatttaatagtttctggggaatattatttaatagtta
tacgctgacggcaccattttgagatttcctcaattaatatttaagaaattgcaaaatgaa
ccatttaacttctaaaagtttctatatacattgattaattacacgacagtccttctcttt
ggcctgtatttcatgctttcacccgtattccctccttatatatagtgacagagttaggtg
ataactcaaaacacaacacaagtttctctctttcctctaattcatcttaacactcaagaa
agtaaagcaggaagtatacaaATGTCGGGAACATTTGACCGTGCCGATGAAGTAAAAGCA
TTCGACGAGATGAAAATCGGTGTGAAGGGTCTCGTCGATGCTGGAATCAAAAAAATCCCA
CTCATATTCCATAACCCGCAAGCCACGGTAACTAACCCTAGACCTCCTTCTACGTTGACT
ATCCCAACAATAGATATGGGAGGTGGCGTGTTCGAGTCCACGGTCACAAGAAAGGAAGTG
ACTGAGAAGGTGAAAGGCGCCATGGAGAAGTTTGGTTTCTTCCAGGCGATAAATCATGGG
ATTCCACTTGAGGTCATGGAGAAGATGAAAGATGGGATCCGTGCGTTTCACGCGCAAGAT
CCAGAAGCGAGGAAAAGGTTCTATAGCCGTGAAAAAACCAAGGCGATTAAGTATAACTCT
AACTCTGATCTCTATGACTCTCCTGCTGCGAGCTGGAGAGATACCTTAAGTTGTTTTATG
TTCCCTGATGTTCCCAAAACCGATGACTTACCAGACATTTGTAGGTACGAACACGGCTTC
TCTTGTTTTAAATTAATCTATGTGCTAACTTAATGTTTTTCTTTGATGGGTTTTGAGAGT
TTATTTATGTTTGAATGTTTTAGGGAGATCATGTTGGACTACTCAAAGAGAGTGATGATG
TTTGGGGAGTTAATCTTTGAGCTCATATCAGAATCCTTAGGGCTGAAGCCTAACCACCTC
AAAGAAATGGATTGTGCAAAAGGTTTGTTGATGCTTTGTCATTGCTACCCGCCGTGTCCT
GAGCCAGACCGAACACTCGGTGCCACTCAGCATACAGACAGATCTTTCATCACTATTCTT
CTTCAAGACCACATTGGAGGACTTCAAGTTCTCCATGATGGATATTGGATCGATGTTCCT
CCTAATCCGAATGCTCTTATCCTTAATGTTGGTGATCTACTACAGGCAAGTTGTTTACAC
ATAATCAAACGGTCCATAAGTAAGAAAACACGGTCTCATATGTTCCAATTGATGACCATG
TCGAAGTAAAAAATTTATAGTGAATTCTTTGTTCCTTAACATTTATACCAGTTATGTTGA
ATTTTTGGTCAAATTATTGATAGGCGGATTCCACTATTTTCCTCTAAATTTTTCATGGAG
AATGAATTTATAAAGAAAAGTTTTCCTCTTCAACTCTGATGAGGAACAAAATGAACAAAA
ATACATACTTTTTATTTTTTCAGTTCTTCAAGTAAAAAATTATTTTTCATTTTGTTCATT
TTTTCTATTCATTTTGTGTTTTTTTTTCAGCTTATAACGAATGACAAGTTTGTGAGTGTG
GAGCATAGAGTTTTGGCAAATGGAGGTGAAGAGCCGCGCACTTCGATTGCATCTTTCTTT
GTGCATCCTCCTTCAACAAATCCGAGAGTATATGGACCGATTAAAGAGCTTTTGTCAGAA
GAAAACCCTCCCAAGTACAGGGAAACCACTCCGGAAGCCTCCAACCACTATGTGGCTAGA
AAACGTGATGGGAACAATTCGTTGAGCCATTTAAGGATCTGA

>BocODD2 DNA sequence:

ATGTCGGGAACATTTGACCGTGCCGATGAAGTAAAAGCATTCGACGAGATGAAAATCGGTGTGAAGGGTCT
CGTCGATGCTGGAATCAAAAAAATCCCACTCATATTCCATAACCCGCAAGCCACGGTAACTAACCCTAGACCT
CCTTCTACGTTGACTATCCCAACAATAGATATGGGAGGTGGCGTGTTCGAGTCCACGGTCACAAGAAAGGAA
GTGACTGAGAAGGTGAAAGGCGCCATGGAGAAGTTTGGTTTCTTCCAGGCGATAAATCATGGGATTCCACT
TGAGGTCATGGAGAAGATGAAAGATGGGATCCGTGCGTTTCACGCGCAAGATCCAGAAGCGAGGAAAAG
GTTCTATAGCCGTGAAAAAACCAAGGCGATTAAGTATAACTCTAACTCTGATCTCTATGACTCTCCTGCTGCGA
GCTGGAGAGATACCTTAAGTTGTTTTATGTTCCCTGATGTTCCCAAAACCGATGACTTACCAGACATTTGTAG
GTACGAACACGGCTTCTCTTGTTTTAAATTAATCTATGTGCTAACTTAATGTTTTTCTTTGATGGGTTTTGAGA
GTTTATTTATGTTTGAATGTTTTAGGGAGATCATGTTGGACTACTCAAAGAGAGTGATGATGTTTGGGGAGTT
AATCTTTGAGCTCATATCAGAATCCTTAGGGCTGAAGCCTAACCACCTCAAAGAAATGGATTGTGCAAAAGG
TTTGTTGATGCTTTGTCATTGCTACCCGCCGTGTCCTGAGCCAGACCGAACACTCGGTGCCACTCAGCATACA
GACAGATCTTTCATCACTATTCTTCTTCAAGACCACATTGGAGGACTTCAAGTTCTCCATGATGGATATTGGAT
CGATGTTCCTCCTAATCCGAATGCTCTTATCCTTAATGTTGGTGATCTACTACAGGCAAGTTGTTTACACATAAT
CAAACGGTCCATAAGTAAGAAAACACGGTCTCATATGTTCCAATTGATGACCATGTCGAAGTAAAAAATTTAT
AGTGAATTCTTTGTTCCTTAACATTTATACCAGTTATGTTGAATTTTTGGTCAAATTATTGATAGGCGGATTCCA
CTATTTTCCTCTAAATTTTTCATGGAGAATGAATTTATAAAGAAAAGTTTTCCTCTTCAACTCTGATGAGGAAC
AAAATGAACAAAAATACATACTTTTTATTTTTTCAGTTCTTCAAGTAAAAAATTATTTTTCATTTTGTTCATTTT
TTCTATTCATTTTGTGTTTTTTTTTCAGCTTATAACGAATGACAAGTTTGTGAGTGTGGAGCATAGAGTTTTGG
CAAATGGAGGTGAAGAGCCGCGCACTTCGATTGCATCTTTCTTTGTGCATCCTCCTTCAACAAATCCGAGAG
TATATGGACCGATTAAAGAGCTTTTGTCAGAAGAAAACCCTCCCAAGTACAGGGAAACCACTCCGGAAGCC
TCCAACCACTATGTGGCTAGAAAACGTGATGGGAACAATTCGTTGAGCCATTTAAGGATCTGA

>BocODD2 cDNA sequence:

ATGTCGGGAACATTTGACCGTGCCGATGAAGTAAAAGCATTCGACGAGATGAAAATCGGTGTGAAGGGTCT
CGTCGATGCTGGAATCAAAAAAATCCCGCTCATATTCCATAACCCGCAAGCCACGGTAACTAACCCTAAACCT
CCTTCTACGTTGACTATCCCAACAATAGATATGGGAGGTGGCGTGTTCGAGTCCACGGTCACAAGAAAGGAA
GTGACTGAGAAGGTGAAAGGCGCCATGGAGAAGTTTGGTTTCTTCCAGGCGATAAATCATGGGATTCCACT
TGAGGTCATGGAGAAGATGAAAGATGGGATCCGTGCGTTTCACGCGCAAGATCCAGAAGCGAGGAAAAG
GTTCTATAGCCGTGAAAAAACCAAGGCGATTAAGTATAACTCTAACTCTGATCTCTATGACTCTCCTGCTGCGA
GCTGGAGAGATACCTTAGGTTGTTTTATGTTCCCTGATGTTCCCAAAACCGATGACTTACCAGACATTTGTAG
GGAGATCATGTTGGACTACTCAAAGAGAGTGATGATGTTTGGGGAGTTAATCTTTGAGCTCATATCAGAATC
CTTAGGGCTGAAGCCTAACCACCTCAAAGAAATGGATTGTGCAAAAGGTTTGTTGATGCTTTGTCATTGCTA
CCCGCCGTGTCCTGAGCCAGACCGAACACTCGGTGCCACTCAGCATACAGACAGATCTTTCATCACTATTCTT
CTTCAAGACCACATTGGAGGACTTCAAGTTCTCCATGATGGATATTGGATCGATGTTCCTCCTAATCCGAATG
CTCTTATCCTTAATGTTGGTGATCTACTACAGCTTATAACGAATGACAAGTTTGTGAGTGTGGAGCATAGAGTT
TTGGCAAATGGAGGTGAAGAGCCGCGCACTTCGATTGCATCTTTCTTTGTGCATCCTCCTTCAACAAATCCG
AGAGTATATGGACCGATTAAAGAGCTTTTGTCAGAAGAAAACCCTCCCAAGTACAGGGAAACCACTCCGGA
AGCCTCCAACCACTATGTGGGTAGAAAACGTGATGGGAACAATTCGTTGAGCCATTTAAGGATCTGA

>BocODD3 promoter sequence:

attgggttccggggttcttattgggtttaggatctagtattttgggtttagggttttggg
ttcaggacattggatttatcgtttgggttaagcgtttattattaagggtttcaggtgtag
catttttggtgtggttatctaaatccttataccaacacacttattcccaaccctacccc
tatacctccacgtaagtgtaaacaagttatctacaaaactgatttcttatacaggtatgt

atcttgttaaccggataacttgtccgttcaccctaagcgataatgtattgaagttcatct
tagcctagtattgttagaattactcgtcggttgtaaatattgaccggtcatagagtttga
ctccatattacaggcacataagtttataagaatcaagatcataactatgacatatgaaat
gaatatacgagtaatgtttaggcaatgttttcattgatcacgacaaattgttagacaatt
aatattatatatatttctttaaaaatgagttacttgagattaaaatccgatttttaatta
atgtcaaattgttatagagtcaatatagtatttacgcggttaacgaataagaagatctta
tatgacacgtcaactgtacaacatatataacatatatatgaagtggagaaacatcggtga
agaacttcgtcggagacgcagcggcggagggcttcactggagacgctgggtgcaagagct
tgaccagggacacagagatcgagaacttcaccggagacgcagcggcggagagttacactg
gagacgcagcggcggagagcttcaccggagacgcagcggaggagagattcaccaaagatg
cagcagccgtctgcgttccatctttctcccccatactgaccgtcacggtgattggttttg
actggtttcgctgaatactagaacaaaatgagagagaaatcccaaatcttaaatttttgt
ttttttaaattctatcgtaatgcagtgaaatgaaatgcaacctgacaaaagatacagaag
aaaataacatgacagagtgatagatcttttcgtatcctaatagaagatttggtctacgat
tttagcggataatgagatatttgtgctgtaggggtagcagagtaaaaaagcacttcgaac
agtgacaattttgattcaatgggtatatcgctaaatacgtaaaaatcttttggtatagct
cctaatttctctaaaatttaactatataacatagaaaaatacttaaatatgatactttct
aaatttgtattgaaaaagtattgaaaccttaatattttaattttgaaatgtccattcaaa
aaatcgcacattagaatatactatatatctatgtccatgtcattaaaatttagttatata
ccatataaataaataaaatgattgttttgatttatttaccaaaacaatatcataaataaa
taagatgtattgttttgatttatgtatttactctaatttagttatatacttaatatgtaa
ataaatacaataaataataatagataaaaattagttttatatataacatttatcccgcgc
aattgcgcaagtctttaacctagttactttgtaaaacacattcttgagattgcattttta
cttgttactgctgacacttataattttaaaatcacatcaattccaaatatgcaccgcact
atggttgctgacatttataacttcaaaacgatatcaattccaaatatgcactgcactatg
gtttccgttcataacctaaaagatacacatctcgatgatatgataataatttttaataat
atgatataaaattgataatatcttttatgatattaataatacagacagtatctcaatata
tctccttctctttgcgctctatttcatgccttcaccagttttccctccttatatatagag
actgcagagctaggtgactcaaaacacaagtttccctgtttcataaagcactaaaaaaaa
ggttggagtccaagtgtacaaATGGTGGGAACATTTAACCGTGCCGGCGAGGTACAAGCA
TTCGACGTACGAGATGAAAATCGGCGTGAAGGGTCTCGTCGACGCTGGAATCACAAAAAT
CCCACTCATGTTCCATAACCTGCAAGCCACGGTAACTAACGCTAAACCTCCTTCTACGTT
GACTATCCCAACAATAGATCTCGGAGGTGGCGTGTTCGAGTCCACGGTCACAAGAAAGGA
AGTGGCCAAGAAGGTTAAAGACGCCATGGAGAAGTTCGGTTTCTTCCAGGCGATCAACCA
TGGGATTCCACTCGAGGTCATGGAGAAGATGAAAGAAGGGATCCGTGCGTTTCACGAGCA
AGATCCAGAAGCGAGGAAAAGGTTCTATAGCCGTGAAAAAACCAAAGCGATTAAGTATAA
CTCTAACTCTGATCTCTATGACGCTCCTGCTGCGAGCTGGAGAGATACCTTAAGTTGTTT
TATGTTCCCTGATGTTCCCAAAACCGATGACTTACCAGACATTTGTAGGTACGAACACGG
CTTCTCTTGTTTTAAATTAATCTTTGGACTAACTTAATATTTTTGTTTGATGGGTTTTGA
GAGTTTATTTATGTTCAATGTTTTAGGGAAATCATGTTGGACTACTCAAAGAGAGTGATG
ATGTTTGGGGAGTTAATATTTGAGCTCATATCAGAATCCTTAGGGCTGAAGCCTAACCAC
CTCAAAGAAATGGATTGTGCAAAAGGCTTGTTGATGCTCTGTCATTGTTACCCGCGGTGT
CCTGAGCCAGACCTAACGCTCGGCGCCACTCAGCATACAGACAGATCTTTCATCACTATA
CTTCTTCAAGACCATATTGGAGGACTTCAAGTTCTCCATGATCGATGGATACCGGATCGA

TGTTCCTCCTAATCCTAATGCTCTTATCCTTAATGTTGGAGATCTCCTACAGGCAAGTTG
TTGTTTACTCATAATCAAACGGTCCATAATTAGTAAGAATACACGGTCTTCATATATATA
TATATATATTTTTTTTTTTTTTTTGCAGCTTATAACGAACGACAAGTTTGTGAGTGTGGAG
CATAGAGTTTTGGCAAATGGAGGTAAAGAGCCACGCACTTCGGTTGCATCTTTCTTTGTG
CATCCTCCTTCAACAAGTCCGAGAGTTTATGGACCCATTAAAGAGCTTTTGTCAGAAGAA
AACCCTCCCAAGTACAGGGAAACCACTTCGGAAGCCTCCAACCACTATGTGGCTAGAAAA
CGTGATGGGAACAATTCGTTGAGCCATTTAAGGATCTGA
>BocODD3 DNA sequence:
ATGAAAATCGGCGTGAAGGGTCTCGTCGACGCTGGAATCACAAAAATCCCACTCATGTTCCATAACCTGCAA
GCCACGGTAACTAACGCTAAACCTCCTTCTACGTTGACTATCCCAACAATAGATCTCGGAGGTGGCGTGTTCG
AGTCCACGGTCACAAGAAAGGAAGTGGCCAAGAAGGTTAAAGACGCCATGGAGAAGTTCGGTTTCTTCCA
GGCGATCAACCATGGGATTCCACTCGAGGTCATGGAGAAGATGAAAGAAGGGATCCGTGCGTTTCACGAG
CAAGATCCAGAAGCGAGGAAAAGGTTCTATAGCCGTGAAAAAACCAAAGCGATTAAGTATAACTCTAACTCT
GATCTCTATGACGCTCCTGCTGCGAGCTGGAGAGATACCTTAAGTTGTTTTATGTTCCCTGATGTTCCCAAAA
CCGATGACTTACCAGACATTTGTAGGTACGAACACGGCTTCTCTTGTTTTAAATTAATCTTTGGACTAACTTAA
TATTTTTGTTTGATGGGTTTTGAGAGTTTATTTATGTTCAATGTTTTAGGGAAATCATGTTGGACTACTCAAAG
AGAGTGATGATGTTTGGGGAGTTAATATTTGAGCTCATATCAGAATCCTTAGGGCTGAAGCCTAACCACCTCA
AAGAAATGGATTGTGCAAAAGGCTTGTTGATGCTCTGTCATTGTTACCCGCGGTGTCCTGAGCCAGACCTAA
CGCTCGGCGCCACTCAGCATACAGACAGATCTTTCATCACTATACTTCTTCAAGACCATATTGGAGGACTTCA
AGTTCTCCATGATCGATGGATACCGGATCGATGTTCCTCCTAA
>BocODD3 cDNA sequence:
ATGAAAATCGGCGTGAAGGGTCTCGTCGACGCTGGAATCACAAAAATCCCACTCATGTTCCATAACCTGCAA
GCCACGGTAACTAACGCTAAACCTCCTTCTACGTTGACTATCCCAACAATAGATCTCGGAGGTGGCGTGTTCG
AGTCCACGGTCACAAGAAAGGAAGTGGCCAAGAAGGTTAAAGACGCCATGGAGAAGTTCGGTTTCTTCCA
GGCGATCAACCATGGGATTCCACTCGAGGTCATGGAGAAGATGAAAGAAGGGATCCGTGCGTTTCACGAG
CAAGATCCAGAAGCGAGGAAAAGGTTCTATAGCCGTGAAAAAACCAAAGCGATTAAGTATAACTCTAACTCT
GATCTCTATGACGCTCCTGCTGCGAGCTGGAGAGATACCTTAAGTTGTTTTATGTTCCCTGATGTTCCCAAAA
CCGATGACTTACCAGACATTTGTAGGGAAATCATGTTGGACTACTCAAAGAGAGTGATGATGTTTGGGGAGT
TAATATTTGAGCTCATATCAGAATCCTTAGGGCTGAAGCCTAACCACCTCAAAGAAATGGATTGTGCAAAAG
GCTTGTTGATGCTCTGTCATTGTTACCCGCGGTGTCCTGAGCCAGACCTAACGCTCGGCGCCACTCAGCATAC
AGACAGATCTTTCATCACTATACTTCTTCAAGACCATATTGGAGGACTTCAAGTTCTCCATGATCGATGGATAC
CGGATCGATGTTCCTCCTAA

>BocODD4 promoter sequence:
aaatagatggatcttgtgaagcaaagaaaccgtgagagaaggctcatgatcacgaggtct
aagaatgttcatgtcttcctacttaacagcattagattatagcttgttacacaaggtact
cacagagatcaaaggaacagattataaggagataaactcctatgtggtggatgctgctac
agattttcgaaattgaaaatggtctggtcataagaaagaaattaaatacaaataatgtag
taatcatcatatggatcactggataagatgaaagaacagcttcgttcctaagctgattca
tggactgaaacataaagcagctgcatatagtatgtaaaagaaattgatcacgcatgatca
ttgatcttggagttgtataaaagacaatccaatacagtccatatatttgaaattccttgt
gattatactaaaccttaaggaggttagtaggcatagaataaatctatgtgggtgtccgac
caaaagcgtccggcccttcaaactaaatatgtccgactctgtccatctaagggcgtccga
ctcttcagcaaagaatgtctggctcttctgctagacgcgtccgacttttaattctaaaag

gcgtctagcccttcttcttgatcacaggctagtagagacaaaagatcaaccggatacaaa
tgtattgtagtccataagcttgtgaaagccgagatctatgacctaataatatatatttta
gtgtgtgatataatccaccgcaacagaggtcatgaaacaccatcaagagatggataaagg
actacaatgtccgagatagatatggtactgcctaaagcaagaaatatcgatgtccacatg
agagtgttttcggccgcagagccataataagagattataaactcacatcaatgatcattg
atcttggacactcatgagacaagtagtggacatgtatagacgaggtctatgacccagaca
tggatcggccagatcgagacataggttcatgataaccatgtctaatacattgtccataag
tacttattttgttcaatcaaagtaaagagttaacagtagacgatcacgtctagactatgg
acacatgcaaacacgatttgcaaagacccaatagtgatccccgagaacaatatggttcac
attattgagcacacatgctttaccgggacactcgatgtcaagagacatgagaaacgacct
aagaggtcaaagtggtctagatacggcttagcaatgaacttggccgattactcccttcct
acatatacaggaaagatcacgcaccagatgcgtagaatgtagaacctacagtgaggttca
catcagggggagtagtgcgtgttgtactctttttccttcatcatggttttgtcccattgg
gtttcctgataaggttttaatgagacaacatgaagcgtactacaaatcctgtatggttat
gacatccaagggggagtgttataaatcatggaatggattgccattaaccatgcccagacc
gagaccggtccaatacctagaagattgagagatggccgaagcctagagagaggagagaga
gaaccgacttcaggagagagagaggcggccacaaagcttggagaaaaggaaaccctttac
ttttcctagtagatgtaatctttctattattatgtattagtagtttttctaatcttagtg
aatttaggttttggatacttttcatttatcttcatcttgtaatccttatataaaagaatc
tttttgatcattaataaacacaaaaatattcggtctccgaatactctatttacaacattt
cttttatttttaatcagtttttgtttgaaaagtcgaagagataaaagtggggtagttcga
gctccaccatcttccgataaggcgataacgacaacaaagctcacgtaagccttgaacacc
gtattctgatcacctaagtattgtaagaatcttcttgtcttttgtctcttttaaaagctt
tgtctcttcgtcctgtttctgGAACAAACACAAACACAAACACAAAAAGGGAGGAAGAAG
AGGATGGAAGCGAAGTACGATCGTGCTACCGAGGTAAAAGCTTTCGATGAAATGAAACTC
GGAGTGAAAGGTCTCGTCGACGCCGGAATCTCAGAGATCCCCCGTATCTTCCACCACCCT
CATCTCACTTCAACGGCTCCAAACCCACTGCTTCCCTCCTCAACGATGATGATCCCAACG
ATCGATCTCGGAGGTGGCGTGTTCGACTCCACAGTCACGCGAGAGAGCGTGATCCCGAAG
ATCAAAGAAGCGGTGGAGAGATACGGGTTCTTCCAGGCGATAAACCATGGGATTCCGGTT
GAAGTTATGGACAAGATGAGAGATAGGGTTTGTGGGTTTCATGAACAAGACTCAGACGTG
AGGAAGAAGCTCTATACCCGGGATAACACCAAGAAGGTTACTTATAATAGTAACTTCGAT
CTCTATAGCTCTCCATCTGCCAACTGGAGAGATACTTTAAGTTGTTTCATGTCCCCTGAT
GTTCCAAGAACAGAAGACTTGCCAGAGATTTGTGGGTAAGAATTAAGTAGATTCGGTTTT
AATATGGTAACTATCTTTTAGTATAAACCGAAATATCGAAAAACTGAAGTAAATATACTT
CTGTATTAAAAAAAAAGGGTAGCTAGGTATACTTCTAATATGGTAGTTAATTTTATAAAC
CAACATATCAAAAAAACTAAAATTAAAACAGAGCTAAAACCAGACCAGACCGACTCCTAG
TAAGAATCATAAAATCTGAACTTGTTTTTTTTTTTTTACATTATTGATGGATTTTGTAAG
TTGTATTGGTCTAAATGTTTTAGGGAGATCATGTTGGAGTATTCAAAGCAAGTGATGGAG
TTAGGGGAGCTAATCTTTGAGCTTTTATCAGAAGCTTTAGGGATGGATCCTAACCATTTG
AAAGAAATGGATTGCACAAAGGGTTTGTTAATGCTCTCTCACTACTACCCGCCTTGTCCT
GAGCCTGACCTAACGTTCGGAACAAGTCAGCACTCAGATAGATCTTTTCTCACTATTCTT
CTTCAACACCACATCGGTGGGCTTCAAGTTCTTCAGGATGGATGTTGGGTGGATGTTCCT
CCTGTTACTCGAGCTCTTCTCATCAACCTTGGAGATTTCTTACAGGTAATTAGTTTTATT
ATATTGACATGATCAATATATCAAACCATTGATTAATACTCTTTTTGTGATTTTTTTTTG

GTTACAGCTTATAACGAATGACAAGTTTTTGAGCGTGGAGCATAGGGTTTTGGCTAACAG
AGGTGACGAGCCTCGTGTTTCGGTAGCATCATTCTTTGTGCATCCTTTGCCGAGTTTTCG
AGTATATGGACCGATGAAAGAGCTTTTGTCTGAACAAAATCCTCCCAAGTATAGAGACAC
TACCGTCACTGAGTACACGAGACATTACATGGCCAGAGGGCTTGATGGGAACTCTGTGTT
ACATCAATTCAAGATCTGATCAAAGCTTTGGACAGACTTACGTATCCAAAGTCTTCTAGG
CAATTTCAGATTAGTATGTAGCATGCTGAAACAAATACACCTGCCTCGGCCAGGACTTCC
AATTGAAACGAGGAGCTTGGACAGGTAGCTATGATGATTCCATCTCGCATATTCCCATGC
AAGATTTTGTTGCTTGTATGATATACATATCCGTTTTGTTGGTAGTATTTTGATTGTTTC
TAGATCATTTGTCAAACGTAAGATTGTATATATAATGCTTTAAAGACGAGATTATTAGTG
AATATCTAATGTTTTATCATGAAGTCAAGTCACAGATGACAATGTCTGCACGGATTTACA
AGATGAGGAGATTAAAGTGAAGACTGTCTCGCTCAATAATGTTATAAGTTAAATGAGAGA
TTATTGTCTAGTAAAAAAACAGAATAAAACATGTTCTATAAGGTGTTGCAAGCTTCAT

>BocODD4 DNA sequence:
ATGGAAGCGAAGTACGATCGTGCTACCGAGGTAAAAGCTTTCGATGAAATGAAACTCGGAGTGAAAGGTCT
CGTCGACGCCGGAATCTCAGAGATCCCCCGTATCTTCCACCACCCTCATCTCACTTCAACGGCTCCAAACCCA
CTGCTTCCCTCCTCAACGATGATGATCCCAACGATCGATCTCGGAGGTGGCGTGTTCGACTCCACAGTCACG
CGAGAGAGCGTGATCCCGAAGATCAAAGAAGCGGTGGAGAGATACGGGTTCTTCCAGGCGATAAACCATG
GGATTCCGGTTGAAGTTATGGACAAGATGAGAGATAGGGTTTGTGGGTTTCATGAACAAGACTCAGACGTG
AGGAAGAAGCTCTATACCCGGGATAACACCAAGAAGGTTACTTATAATAGTAACTTCGATCTCTATAGCTCTC
CATCTGCCAACTGGAGAGATACTTTAAGTTGTTTCATGTCCCCTGATGTTCCAAGAACAGAAGACTTGCCAG
AGATTTGTGGGTAAGAATTAAGTAGATTCGGTTTTAATATGGTAACTATCTTTTAGTATAAACCGAAATATCGA
AAAACTGAAGTAAATATACTTCTGTATTAAAAAAAAAGGGTAGCTAGGTATACTTCTAATATGGTAGTTAATTT
TATAAACCAACATATCAAAAAAACTAAAATTAAAACAGAGCTAAAACCAGACCAGACCGACTCCTAGTAAGA
ATCATAAAATCTGAACTTGTTTTTTTTTTTTTACATTATTGATGGATTTTGTAAGTTGTATTGGTCTAAATGTTTT
AGGGAGATCATGTTGGAGTATTCAAAGCAAGTGATGGAGTTAGGGGAGCTAATCTTTGAGCTTTTATCAGA
AGCTTTAGGGATGGATCCTAACCATTTGAAAGAAATGGATTGCACAAAGGGTTTGTTAATGCTCTCTCACTAC
TACCCGCCTTGTCCTGAGCCTGACCTAACGTTCGGAACAAGTCAGCACTCAGATAGATCTTTTCTCACTATTC
TTCTTCAACACCACATCGGTGGGCTTCAAGTTCTTCAGGATGGATGTTGGGTGGATGTTCCTCCTGTTACTCG
AGCTCTTCTCATCAACCTTGGAGATTTCTTACAGGTAATTAGTTTTATTATATTGACATGATCAATATATCAAAC
CATTGATTAATACTCTTTTTGTGATTTTTTTTTGGTTACAGCTTATAACGAATGACAAGTTTTTGAGCGTGGAG
CATAGGGTTTTGGCTAACAGAGGTGACGAGCCTCGTGTTTCGGTAGCATCATTCTTTGTGCATCCTTTGCCG
AGTTTTCGAGTATATGGACCGATGAAAGAGCTTTTGTCTGAACAAAATCCTCCCAAGTATAGAGACACTACC
GTCACTGAGTACACGAGACATTACATGGCCAGAGGGCTTGATGGGAACTCTGTGTTACATCAATTCAAGATC
TGA
>BocODD4 cDNA sequence:
ATGGAAGCGAAGTACGATCGTGCTACCGAGGTAAAAGCTTTCGATGAAATGAAACTCGGAGTGAAAGGTCT
CGTCGACGCCGGAATCTCAGAGATCCCCCGTATCTTCCACCACCCTCATCTCACTTCAACGGCTCCAAACCCA
CTGCTTCCCTCCTCAACGATGATGATCCCAACGATCGATCTCGGAGGTGGCGTGTTCGACTCCACAGTCACG
CGAGAGAGCGTGATCCCGAAGATCAAAGAAGCGGTGGAGAGATACGGGTTCTTCCAGGCGATAAACCATG
GGATTCCGGTTGAAGTTATGGACAAGATGAGAGATAGGGTTTGTGGGTTTCATGAACAAGACTCAGACGTG
AGGAAGAAGCTCTATACCCGGGATAACACCAAGAAGGTTACTTATAATAGTAACTTCGATCTCTATAGCTCTC
CATCTGCCAACTGGAGAGATACTTTAAGTTGTTTCATGTCCCCTGATGTTCCAAGAACAGAAGACTTGCCAG
AGATTTGTGGGGAGATCATGTTGGAGTATTCAAAGCAAGTGATGGAGTTAGGGGAGCTAATCTTTGAGCTT

TTATCAGAAGCTTTAGGGATGGATCCTAACCATTTGAAAGAAATGGATTGCACAAAGGGTTTGTTAATGCTC
TCTCACTACTACCCGCCTTGTCCTGAGCCTGACCTAACGTTCGGAACAAGTCAGCACTCAGATAGATCTTTTC
TCACTATTCTTCTTCAACACCACATCGGTGGGCTTCAAGTTCTTCAGGATGGATGTTGGGTGGATGTTCCTCC
TGTTACTCGAGCTCTTCTCATCAACCTTGGAGATTTCTTACAGCTTATAACGAATGACAAGTTTTTGAGCGTG
GAGCATAGGGTTTTGGCTAACAGAGGTGACGAGCCTCGTGTTTCGGTAGCATCATTCTTTGTGCATCCTTTG
CCGAGTTTTCGAGTATATGGACCGATGAAAGAGCTTTTGTCTGAACAAAATCCTCCCAAGTATAGAGACACT
ACCGTCACTGAGTACACGAGACATTACATGGCCAGAGGGCTTGATGGGAACTCTGTGTTACATCAATTCAAG
ATCTGA

supplementary data 2 Phylogenetic analysis of ODDs in the Brassica family

>BocODD1
MAGRFDRAGEVKAFDEMKIGVKGLVDNGITKIPRIFHNPQATITNPKPPSTLTIPTIDLGGGVFESTVTRKEVTEK
VKGAMEKFGFFQAINHGIPLEVLEKMKDGIRAFHAQDPEARKRFYSREKTKAIKYNSNSDLYDSPAASWRDTLS
CFMFPDVPKTDDLPDICREIMLDYSKRVMMFGELIFELISESLGLKPNHLKEMDCAKGLLMLCHCYPRCPEPDLT
LGATQHTDRSFITILLQDHIGGLQVLHDGYWIDVPPNPNALILNVGDLLQLITNDKFVSVEHRVLANGGKEPRTS
VASFFVHPPSISPRVYGPIKELLSEENPPKYRETTPEASNHYVARKRDGNNSLSHLRI
>BocODD3
MKIGVKGLVDAGITKIPLMFHNLQATVTNAKPPSTLTIPTIDLGGGVFESTVTRKEVAKKVKDAMEKFGFFQAIN
HGIPLEVMEKMKEGIRAFHEQDPEARKRFYSREKTKAIKYNSNSDLYDAPAASWRDTLSCFMFPDVPKTDDLP
DICREIMLDYSKRVMMFGELIFELISESLGLKPNHLKEMDCAKGLLMLCHCYPRCPEPDLTLGATQHTDRSFITILL
QDHIGGLQVLHDRWIPDRCSS
>BocODD4
MEAKYDRATEVKAFDEMKLGVKGLVDAGISEIPRIFHHPHLTSTAPNPLLPSSTMMIPTIDLGGGVFDSTVTRES
VIPKIKEAVERYGFFQAINHGIPVEVMDKMRDRVCGFHEQDSDVRKKLYTRDNTKKVTYNSNFDLYSSPSANW
RDTLSCFMSPDVPRTEDLPEICGEIMLEYSKQVMELGELIFELLSEALGMDPNHLKEMDCTKGLLMLSHYYPPCP
EPDLTFGTSQHSDRSFLTILLQHHIGGLQVLQDGCWVDVPPVTRALLINLGDFLQLITNDKFLSVEHRVLANRGD
EPRVSVASFFVHPLPSFRVYGPMKELLSEQNPPKYRDTTVTEYTRHYMARGLDGNSVLHQFKI
>BocODD2
MSGTFDRADEVKAFDEMKIGVKGLVDAGIKKIPLIFHNPQATVTNPKPPSTLTIPTIDMGGGVFESTVTRKEVTE
KVKGAMEKFGFFQAINHGIPLEVMEKMKDGIRAFHAQDPEARKRFYSREKTKAIKYNSNSDLYDSPAASWRDT

LGCFMFPDVPKTDDLPDICREIMLDYSKRVMMFGELIFELISESLGLKPNHLKEMDCAKGLLMLCHCYPPCPEP
DRTLGATQHTDRSFITILLQDHIGGLQVLHDGYWIDVPPNPNALILNVGDLLQLITNDKFVSVEHRVLANGGEEP
RTSIASFFVHPPSTNPRVYGPIKELLSEENPPKYRETTPEASNHYVGRKRDGNNSLSHLRI
>AT2G25450
MAENYDRASELKAFDEMKIGVKGLVDAGVTKVPRIFHNPHVNVANPKPTSTVVMIPTIDLGGVFESTVVRESV
VAKVKDAMEKFGFFQAINHGVPLDVMEKMINGIRRFHDQDPEVRKMFYTRDKTKKLKYHSNADLYESPAAS
WRDTLSCVMAPDVPKAQDLPEVCGEIMLEYSKEVMKLAELMFEILSEALGLSPNHLKEMDCAKGLWMLCHCF
PPCPEPNRTFGGAQHTDRSFLTILLNDNNGGLQVLYDGYWIDVPPNPEALIFNVGDFLQLISNDKFVSMEHRIL
ANGGEEPRISVACFFVHTFTSPSSRVYGPIKELLSELNPPKYRDTTSESSNHYVARKPNGNSSLDHLRI
>Bo4g173530.1
MAGRFDRAGEVKAFDEMKIGVKGLVDNGITKIPRIFHNPQATVTNPKPPSTLTIPTIDLGGGVFESTVTRKEVTE
KVKGAMEKFGFFQAINHGIPLEVLEKMKDGIRAFHAQDPEARKRFYSREKTKAIKYNSNSDLYNSPAASWRDTL
SCFMFPDVPKTDDLPDICREIMLDYSKRVMMFGELIFELISESLGLKPNHLKEMDCAKGLLMLCHCYPRCPEPDL
TLGATQHTDRSFITILLQDHIGGLQVLHDGYWIDVPPNPNALILNVGDLLQLLTNDKFGGGGDRVLANGGKEPR
TSVASFFVHPPSISPRVYGPIKELLSEENPPKYRETTPEASNHYVARKRDGNNSLSHLRI
>Bo4g173550.1
MAGRFDRAGEVKAFDEMKIGVKGLVDNGITKIPRIFHNPQATVTNPKPPSTLTIPTIDLGGGVFESTVTRKEVTE
KVKGAMEKFGFFQAINHGIPLEVLEKMKDGIRAFHAQDPEARKRFYSREKTKAIKYNSNSDLYDAPAASWRDAL
SCFMFPDVLKTDDLPDICREIMLDYSKRVKMFGELIFELISESLGLNPNHLKEMDCAKGLLMLCHCYPPCPEPDR
TLGATQHTDRSFITILLQDHIGGLQVLHDGYWIDVPPNPNALILNVGDLLQLITNDKFVSVEHRVLANGGKEPRT
SVASFFVHPPSTSPRVYGPIKELLSEENPPKYRETTSKASNHYVART
>Bo4g173560.1
MSGTFDRADEVKAFDEMKIGVKGLVDAGIKKIPLIFHNPQATVTNPKPPSTLTIPTIDMGGGVFESTVTRKEVTE
KVKGAMEKFGFFQAINHGIPLEVLEKMKDGIRAFHAQDQEARKRFYSREKTKAIKYNSNSDLYDSPAASWRDTL
SCFMFPDVPKTDDLPDICREIMLDYSKRVMMFGELIFELISESLGLKPNHLKEMDCAKGLLMLCHCYPPCPEPD
RTLGATQHTDRSFITILLQDHIGGLQVLHDGYWIDVPPNPNALILNVGDLLQLITNDKFVSVEHRVLANGGEEPR
TSIASFFVHPPSTNPRVYGPIKELLSEENPPKYRETTPEASNHYVARKRDGNNSLSHLRI
>BnaA04g17890D
MSGTFDRADEVKAFDEMKIGVKGLVDAGIKKIPLIFHNPQATVTNPKPPSTLTIPTIDMGGGVFESTVKRKEVTE
KVKDAMEKFGFFQAINHGIPLEVMEKMKDGIRAFHAQDPEARKRFYSREKTKAIKYNSNSDLYDSPAASWRDT
LSCFMFPDVPKTDDLPDICREIMLDYSKRMMMFGELIFELISESLGLNPNHLKEMDCAKGLLMLCHCYPPCPEP
DRTLGATQHTDRSFITILLQDHIGGLQVLHDGYWIDVPPNPNALILNLITNDKFVSVEHRVLANGGEEPRTSIASF
FVHPPSTSPRVYGPIKELLSEENPPKYRETTPEASNHYVARKRDGNNSLSHLRI
>BnaA04g17880D
MAGTFDRADEVKAFDEMKIGVKGLVDAGITKIPRIFHNPQATVTNPKPPSTLTIPTIDLRGGVFESTVTRKEVTEK
FGFFQAINHGIPLEVMEKIKEGIRAFHAQDPEARKRFYSREKTKAIKYNSNSDLYDSPAASWRDTLSCFMFPDVP
KTDDLPDICREIMLDYSKRVMMFGELIFELISESLGLKPNHLKKMDCAKGLLMLCHCYPPCPEPDLTLGATQHTD
RSFITILLQDHIGGLQVLHDGYWIDVPPNPNALILNLITNDKFVSVEHRVLANGGKEPRTSVASFFVHPPSTSPRV
YGPIKELLSEENPPKYRETTPEASNHYVARKRDGNNSLSHLRI-
>BnaA03g15200D
MESKGTLVDMLDRASEVKTFDEMKMGVKGLVEAGMTKIPRIFHNPLASVTTPKPPSTVRIPTIDLRGGVFDSEV
TRQSVVAKVKEAMEKFGFFQAINHGIPLHVMEEMEAGIRGFHGQDPEARKMFYSRDKTKKVKYNSNVDLYDS
PAASWRDTLSLFLAPDVPKAEDLPEICGEIILEYSQGVMKLAELIFELLSESLGLSRNHLKELDCAKGLLMLCHCYP
PCPEPDLTLGATQHTDRSFVTILLQDHIGGLQVLHDGYWIDVPPTPGALILNTGDLLQASCCSQLKIKRFYIIQLIT

NDKFVSVEHRVLANGRDEPRTSVASFFVHPSSSSRVYGPIKELLSEQNPPKYRDTTAEASNHYVPRKGVGNASLS
HLRI-
>BnaA03g15210D
MESKGTLVDMLDRASEVKTFDEMKMGVKGLVEAGMTKIPRIFHNPLASVTTPKPPSTVRIPTIDLRGGVFDSEV
TRQSAMEKFGFFQAINHGIPLHVMEEMEAGIRGFHGQDPEARKMFYSRDKTKKVKYNSNVDLYDSPAASWR
DTLSLFLAPDVPKAEDLPEICGEIILEYSQGVMKLAELIFELLSESLGLSRNHLKELDCAKGLLMLCHCYPPCPEPDL
TLGATQHTDRSFVTILLQDHIGGLQVLHDGYWIDVPPTPGALILNTGDLLQASCCSQLKIKRFYIIQLITNDKFVSV
EHRVLANGRDEPRTSVASFFVHPSSSSRVYGPIKELLSEQNPPKYRDTTAEASNHYVPRKGVGNASLSHLRI
>BnaA04g17870D
MAGLYDRDSEVKAFDEMKIGVKGLVDAGITHIPRIFHHSPHVTVENPIKPSSTVVIPTIDLGGGMFESTVTRENV
VAEVRDAVEKFGFFQVIKHGIPLDVMEKMKEGTRGFHEQDTEVKRGFYSRDITKKVKYNTNFDLYSSQAANWR
DTLTTVMAPDVPRAEDLPKICGYVLITPIGPCIHMACNWKMSISV
>BnaC05g04040D
MESTATIALDRSTQLKAFDETKTGVKGLVEAGISEIPAIFHAPPSTITTPKPPSSSQFTIPTIDLQGGSTDSISRRSLVI
NHGIPLNVMDRMKEGVREFHELDPEVRKGFYSRDSSSSNMLYMSNFDLYNSPAANWRDTLVCFTAPDPPRPE
DLPAACGEVMIEYSNEVMKVGKMLFELLSEALGLNTNHLKDMDCANSLLLLGHYYPPCPQPNLTLGLTKHSDN
SFLTVLLQDHVGGLQVLHDQYWVDVPPVPGALVVNVGDLLQLITNGKFISVEHRVLANGAGPRISVACFFSSY
WMENPRVYGPIKELLSEENPPIYRDTTITEYSKFYRSKGFDGTSGLLYLKI
>BnaA04g17860D
MMKLGELIFGLLSEALGLEPNHLKELDCAKSLSLLSHYYPPCPEPDRTFGISSHTDISFITVLLQDHIGGLQVLHDG
CWINVPPNPKALILISNNKFVSVEHRVLANRSEEPRISIASFFMHTIPNEQVYGPIKELISTQNPPKYRDTTTTELA
RHYLARGLDGASPLLHFRI
>BnaC04g41490D
MSGTFDRADEVKAFDEMKIGVKGLVDAGIKKIPLIFHNPQATVTNPKPPSALTIPTIDMGGGVFESTVTRKEVTE
KFGFFQAINHGIPLEVMEKMKDGIRAFHAQDPEARKRFYSREKTKAIKYNSNSDLYDSPAASWRDTLSCFMFPD
VPKTDDLPDICREIMLDYSKRVMRFGELIFELISESLGLKPNHLKEMDFAKGLLMLCHCYPPCPEPDRTLGATQHT
DRSFITILLQDHIGGLQVLHDGYWIDVPPNPNALILNVGDLLQAICVFFQLITNDKFVSVEHRVLANGGEEPRTSI
ASFFVHPPSTNPRVYGPIKELLSEENPPKYRETTPEASNHYVARKRDGNNSLSHLRI
>BnaCnng70010D
MVGTFNRAGEVQAFDEMKIGVKGLVDAGITKIPLMFHNPQATVTNAKPPSTLTIPTIDLGGGVFESTVTRKEVA
KKVKDAMDKFGFFQAINHGIPLEVMEKMKEGIRAFHAQDPEARKSWRDTLSCFMFPDVPKTDELPDICREIM
LDYSKRVMMFGELIFELISESLGLKPNHLKEMDCAKGLLMLCHCHPPCPQPDLTVGATQHTDRSFITILLQDHIG
GLQVLHDGYWIDVPPNPNALILNLMTNDKFVSVEHRVLANGGKEPRTSVASFFVHPPSTSPRVYGPIKELLSEE
NPPKYRETTPEASNHYVARKRDGNNSLSHLRI
>Bra021670.1
MAGTFDRAGEVKAFDEMKIGVKGLVDAGITKIPRIFHNPQATVTNPKPPSTLTIPTIDMGGCVFESTVARKEVTE
KVKDAMEKFGFFQAINHGIPLEVMEKMKDGIRAFHAQDPEARKRFYSREKTKAIKYNSNSDLYDSPAASWRDT
LSCFMFPDVPKTDDLPDICREIMLDYSKRVMMFGELIFELISESLGLKPNHLKKMDCAKGLLMLCHCYPPCPEPD
RTLGATQHTDRSFITILLQDHIGGLQVLHDGYWIDVPPNPNALILNLITNDKFVSVEHRVLANGGKEPRTSVASFF
VHPPSTSPRVYGPIKELLSEENPPKYRETTPEASNHYVARKRDVNNSLSHLRI
>Bra021671.1
MSGTFDRANEVKAFDEMKIGVKGLVDAGIKKIPLIFHNPQATVTNPKPPSTLTIPTIDMGGGVFESTVARKEVTE
KVKDAMEKFGFFQAINHGIPLEVMEKIKEGIRAFHAQDPEARKRFYSREKTKAIKYNSNSDLYDSPAASWRDTLS
CFMFPDVPKTDDLPDICREIMLDYSKRVMMFGELIFELISESLGLKPNHLKKMDCAKGLLMLCHCYPPCPEPDR

TLGATQHTDRSFITILLQDHIGGLQVLHDGYWIDVPPNPNALILNLITNDKFVSVEHRVLANGGEEPRTSIASFFV
HPPSTSPRVYGPIKELLSEENPPKYRETTPEASNHYVARKRDGNNSLSHLRI

>Bra022920.1

MESKGTLVDMLDRASEVKTFDEMKMGVKGLVEAGMTKIPRIFHNPLASVTTPKPPSTVRIPTIDLRGGVFDSEV
TRQSVVAKVKEAMEKFGFFQAINHGIPLHVMEEMEAGIRGFHGQDPEARKMFYSRDKTKKVKYNSNVDLYDS
PAASWRDTLSLFLAPDVPKAEDLPEICGEIILEYSQGVMKLAELIFELLSESLGLSRNHLKELDCAKGLLMLCHCYP
PCPEPDLTLGATQHTDRSFVTILLQDHIGGLQVLHDGYWIDVPPTPGALILNTGDLLQASCCSQLKIKRFYIIQLIT
NDKFVSVEHRVLANGRDEPRTSVASFFVHPSSSSRVYGPIKELLSEQNPPKYRDTTAEASNHYVPRKGVGNASLS
HLRI

>Bra021669.1

MAGLYDRDSEVKAFDEMKIGVKGLVDAGITHIPRIFHHSPHVTVENPIKHSSTVVIPTIDLGGGMFESTVTRENV
VAEVRDAVEKFGFFQVIKHGIPLDVMEKMKEGTRGFHEQDTEVKRGFYSRDITKKVKYNTNFDLYSSQAANWR
DTLTTVMAHDVPRAEDLPKICGYVLITPIGPCIHMACNWKMSISV

>Bra021668.1

MMKLGELIFGLLSEALGLEPNHLKELDCAKSLSLLSHYYPPCPEPDRTFGISSHTDISFITVLLQDHIGGLQVLHDG
CWIDVPPNPKALILISNDKFVSVEHRVWANRSEEPRISIASFFMHTIPNEQVYGPIKELVSTQNPPKYRDTTTTEL
ARHYLARGLDGASPLLHFRI
